# Conventional HDL Subclass Measurements Mask Thyroid Hormone-dependent Remodeling Activity Sites in Hypothyroid Individuals

**DOI:** 10.1210/jendso/bvae018

**Published:** 2024-02-13

**Authors:** John D Bagdade, Carrie E McCurdy

**Affiliations:** Department of Human Physiology, University of Oregon, Eugene, OR 97403, USA; Department of Human Physiology, University of Oregon, Eugene, OR 97403, USA

**Keywords:** HDL subclasses, hypothyroid, lipoprotein, remodeling proteins

## Abstract

**Context:**

Earlier nuclear magnetic resonance spectroscopy (NMR) studies of plasma lipoproteins estimated by size as small, medium, and large particles, demonstrated hypothyroidism was associated with increases in very low-density lipoprotein (VLDL), low-density lipoprotein (LDL), and intermediate-density lipoprotein (IDL) subclass particle number but variable changes in the high-density lipoprotein (HDL) subclasses. These disparate changes in HDL might be explained by reduced activity of the thyroid hormone-dependent remodeling proteins whose subclass specificity may be obscured when the 5 HDL subclasses identified by NMR are combined by size.

**Objective:**

This work aimed to determine whether directional changes in particle number of individually measured HDL subclasses correlate with reduced activity of their thyroid hormone–dependent remodeling proteins in hypothyroid individuals.

**Methods:**

VLDL, LDL, IDL, and HDL subclasses were measured by NMR in 13 thyroidectomized individuals 1 month following thyroid hormone withdrawal and 3 months after replacement. Changes in particle numbers in each subclass were compared when expressed individually and by size.

**Results:**

Following thyroid hormone withdrawal, plasma lipids and VLDL, LDL, and IDL subclass particle number increased. HDL particle number nearly doubled in very small HDL-1 (*P* = .04), declined in small HDL-2 (*P* = .02), and increased 2-fold in HDL-5 (*P* = .0009).

**Conclusion:**

The increment in HDL-1 and decline in HDL-2 subclasses is consistent with their precursor-product relationship and reduced lecithin cholesterol acyltransferase activity while the almost 2-fold increase in large HDL-5 is indicative of diminished action of hepatic lipase, phospholipid transfer protein, and endothelial lipase. These findings are inapparent when the 5 subclasses are expressed conventionally by size. This linking of specific HDL subclasses with HDL remodeling protein function provides new details about the specificity of their interactions.

Measuring plasma lipoprotein particle numbers by nuclear magnetic resonance spectroscopy (NMR) is less time-consuming and labor intensive than other previously employed methods of lipoprotein quantitation and is one of several methods applied clinically for risk assessment [1]. Lipoproteins measured by NMR separate into 15 subclasses: 6 in very low-density lipoprotein (VLDL), 3 in low-density lipoprotein (LDL), 1 in intermediate-density lipoprotein (IDL), and 5 in high-density lipoprotein (HDL) [2]. To simplify reporting, the subclasses in each density fraction by convention have been combined according to size and reported as small-, medium-, and large-sized particles. This method of data analysis reduces the number of subclasses in VLDL, LDL, and IDL from 10 to 7 and in HDL from 5 to 3.

In an earlier study, Pearce et al [3] examined the relationship between these subclasses grouped by size and markers of thyroid function in 28 women with short-term hypothyroidism. Consistent with the increase in plasma triglycerides (TGs), total cholesterol (TC), and LDL lipids observed clinically when thyroid hormone is deficient [4], these researchers observed that all the VLDL and 2 of the 3 LDL subclasses and IDL were elevated. Additionally, however, they found a very different pattern of change in HDL wherein small and mid-sized HDL particles both declined and large HDL particles and HDL cholesterol (HDL-C) increased. Considering that the transport of HDL is more complex than that of the apolipoprotein B (apoB)-containing lipoproteins, this difference in the directional responses of their subclasses is not surprising.

The transport of the apoB lipoproteins, for example, requires the coordinated actions of several thyroid hormone–dependent systems remodeling proteins: (1) LPL, which converts a portion of TG-rich lipoproteins (TGRLPs) to LDL and IDL [5]; (2) cholesteryl ester transfer protein (CETP), which functions in the heteroexchange of core lipids between HDL and TGRLPs [6]; (3) phospholipid transfer protein (PLTP), which facilitates the movement of lipoprotein surface and core lipids between TGRLPs and HDL [7], and; (4) hepatic lipase (HL), which remodels large HDL and degrades TGRLP remnants [8]. The net effect of the actions of these 4 remodeling proteins is to decrease the size of the apoB lipoproteins and facilitate their exocytotic removal by tissue apoB, E receptors, which also are thyroid hormone dependent [9].

In contrast, HDL transport involves the actions of 6 remodeling proteins that together simultaneously generate and enlarge new particles and downsize those that are large and mature. These events require the coordinated action of the same 4 apoB lipoprotein processing proteins (discussed earlier) and 2 others that act solely on HDL: (1) lecithin cholesterol acyltransferase (LCAT), which initiates the formation of new HDL particles by esterifying tissue-derived free cholesterol on nascent HDL discs (HDL-1) [10] and is thyroid hormone dependent; and (2) endothelial lipase (EL), whose triglyceridase and phospholipase actions downsize large HDL-4 and HDL-5 particles [11]. EL is the only lipoprotein remodeling protein that has not yet been shown to be regulated by thyroid hormone [12].

No prior attempt has been made to relate dysfunction of these thyroid hormone–dependent HDL remodeling proteins to changes in particle number in hypothyroid patients when each of the 5 HDL subclasses is expressed individually. To obtain this information and further details about the specificity of the remodeling proteins that was not apparent in the Pearce report [3] in which the HDL subclasses were estimated according to size, we have examined lipoproteins by NMR in individuals with short-term hypothyroidism.

## Materials and Methods

### Study Participants

Thirteen otherwise healthy individuals who had previously undergone subtotal thyroidectomy for thyroid cancer (3 men and 10 women; mean age 45.7 ± 10.6 years ± SD) were studied. All participants were selected from a population of patients followed at the Cook County Hospital Thyroid Clinic over a 9-month period in 1993 to 1994. None had diabetes based on fasting glucose, hypertension (blood pressure < 140/90) or renal disease based on normal blood urea nitrogen and serum creatinine or was taking a medication that affected lipid metabolism. The protocol was approved by the institutional review board of the John H. Stroeger, Jr. Hospital of Cook County and written informed consent was obtained. Blood samples (EDTA) were obtained on 2 occasions after an overnight (14-hour) fast. The first sample was drawn 4 weeks after each participant had discontinued their maintenance dose of levothyroxine (0.183 ± 0.033 mcg day dose mean ± SD) in preparation for diagnostic thyroid scanning. The second blood sample was obtained 3 months after levothyroxine treatment was resumed. Aliquots of plasma from each sample were frozen and stored at −80 °C and not thawed until the time measurements were performed. Total thyroxine (T4) was measured by radioimmunoassay (Diagnostic Products) using a coated-tube technique. The minimal detectable concentrations of T4 in the assay was 0.25  μg. Thyrotropin (TSH) was measured by an immunoradiometric technique (Allegro Highly Sensitive TSH; Nichols Institute). The lowest limit of detection for this method is 0.03 units. All hormone assay analysis occurred in 1995. Hormone measures and plasma lipids in these individuals (Table 1) have been previously published [6, 13].

**Table 1.** Effects of thyroid hormone withdrawal and replacement on thyroid function and plasma lipids

Measurement	Withdrawal	Treatment	*P*
Triglycerides	126.1 ± 41.9	80.5 ± 27.9	.003
Total cholesterol	200.4 ± 46.2	148.9 ± 39.7	.005
LDL-C	131.1 ± 41.7	95.4 ± 35.6	.027
HDL-C	51.2 ± 13.7	42.8 ± 10	.087

Data are presented as the mean ± SD. Data were analyzed using paired *t* tests.

Abbreviations: HDL-C, high-density lipoprotein cholesterol; LDL-C, low-density lipoprotein cholesterol.

### Measurement of Lipids and Lipoprotein Subclasses

All lipid measurements including quantitation of the number of particles within each lipoprotein subclass by NMR were performed at Liposcience, Inc, as previously described [2]. These measurements were all performed on frozen plasma within 1 year of sample collection (average frozen storage time 2.7 months). Each NMR measurement includes the concentrations of 6 subclasses of VLDL (VLDL 1-6; larger numbers denoting larger subclasses), 3 subclasses of LDL (LDL 1-3) and intermediate density lipoproteins (IDL), and 5 subclasses of HDL (HDL1-5), as well as calculated mean particle sizes of VLDL, LDL, and HDL. Measurements of the lipoprotein subclasses following thyroid hormone withdrawal and replacement were expressed in 2 ways: (1) by size as small, medium, and large particles, and (2) as individual subclasses within each density fraction.

### Statistical Analyses

Data from participants obtained while hypothyroid and after replacement with thyroid hormone expressed as individual and combined subclasses were analyzed and compared using a paired *t* test with Holms-Sidak multiple test correction. In this secondary analysis, all patients were included from the prior studies examining the effect of thyroid hormone on aspects of lipid metabolism [6, 13]. These primary studies were powered to detect a medium effect size with α = 0.05% and 80% power for main outcomes in lipoprotein subclasses.

## Results

Hypothyroidism after hormone withdrawal was confirmed by low serum T4 and elevated TSH concentrations as previously published [13]. When the study participants were hypothyroid, plasma TGs, TC, and LDL cholesterol (LDL-C) were all significantly higher than those found after their T4 levels returned to normal (see Table 1).

### Lipoprotein Subclasses Measured Individually Very Low-Density Lipoprotein

**Figure 1.**
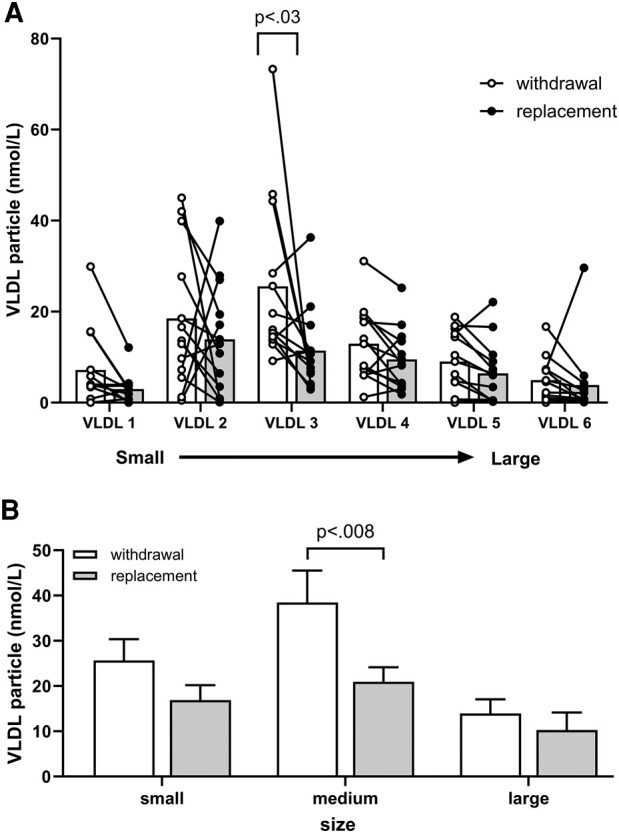
Effects of thyroid hormone withdrawal (open bars) and replacement (shaded bars) on very low-density lipoprotein (VLDL) subclasses measured by nuclear magnetic resonance spectroscopy (mean ± SD). A, Particle concentrations of lipoproteins were calculated for 6 subclasses. B, Combined subclass particle concentration into small, medium, and large sizes. Data were analyzed by paired *t* test with Holm-Sidak multiple test correction.

When thyroid hormone was withdrawn, the mean particle number increased in all 6 VLDL subclasses (Table 2 and Fig. 1A). The magnitudes of these increases were relatively greater in the smaller sized particles (ie, VLDL-1, VLDL-2, and VLD-3) and the increment (+125%) in VLDL-3 was statistically significant (*P* = .03). When the particles are combined into small, medium, and large particles, the medium-sized particle remains statistically different after treatment (Fig. 1B).

**Table 2.** Effect of thyroid hormone withdrawal and replacement on lipoprotein subclass particle number

NMR subclass	Withdrawal	Replacement	Adjusted *P*
VLDL-1	7.2 ± 8.5	3.0 ± 3.1	NS
VLDL-2	18.5 ± 15.4	13.9 ± 12.2	NS
VLDL-3	25.6 ± 18.5	11.4 ± 9.1	.03
VLDL-4	12.9 ± 8.2	9.5 ± 6.7	NS
VLDL-5	9.0 ± 6.8	6.4 ± 6.8	NS
VLDL-6	4.9 ± 4.8	3.9 ± 7.9	NS
Average size	49.9 ± 6.8	50.2 ± 8.9	NS
LDL-1	15.1 ± 15.4	12.8 ± 7.9	NS
LDL-2	40.4 ± 24.7	28.9 ± 14.8	NS
LDL-3	62.6 ± 25.7	47.5 ± 23.1	NS (.06)
Average size	25.9 ± 0.3	25.8 ± 0.2	NS
IDL	13.0 ± 11.1	6.2 ± 4.6	.01
HDL-1	5.0 ± 2.6+	2.6 ± 2.4	.04
HDL-2	3.5 ± 4.1	7.0 ± 4.7	.02
HDL-3	7.5 ± 4.2	9.6 ± 3.8	NS
HDL-4	15.0 ± 7.1	13.7 ± 6.7	NS
HDL-5	20.2 ± 6.5	9.7 ± 4.8	.0009
Average size	10.0 ± 0.3	9.4 ± 0.3	.0001

Data are presented as the mean ± SD. Data were analyzed using paired *t* tests with the Holms-Sidak method for multiple comparisons within a lipid class where appropriate.

Abbreviations: HDL, high-density lipoprotein; LDL, low-density lipoprotein; NS, not significant; VLDL, very low-density lipoprotein.

### Intermediate-Density Lipoprotein

**Figure 2.**
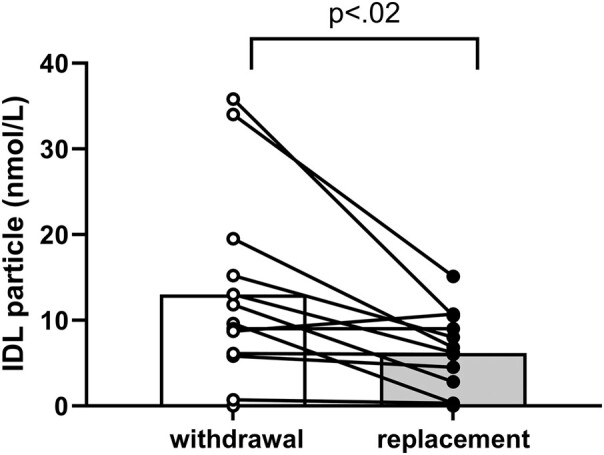
Effects of thyroid hormone withdrawal (open bars) and replacement (shaded bars) on intermediate-density lipoprotein measured by nuclear magnetic resonance spectroscopy (mean ± SD). Data were analyzed by paired *t* test.

The number of IDL particles also increased (+110%) and was significantly higher (see Table 2; *P* = .01) when thyroid hormone was withheld (Fig. 2).

### Low-Density Lipoprotein

**Figure 3.**
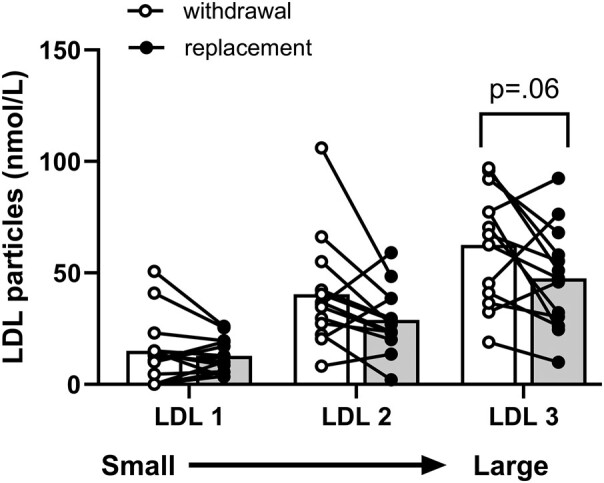
Effects of thyroid hormone withdrawal (open bars) and replacement (shaded bars) on low-density lipoprotein subclasses measured by nuclear magnetic resonance spectroscopy (mean ± SD). Data were analyzed by paired *t* test with Holm-Sidak multiple test correction.

During the interval of thyroid hormone withdrawal, particle number increased in all 3 LDL subclasses (see Table 2 and Fig. 3) and approached statistical significance in large LDL-3 (+32%; *P* = .06).

### High-Density Lipoprotein

**Figure 4.**
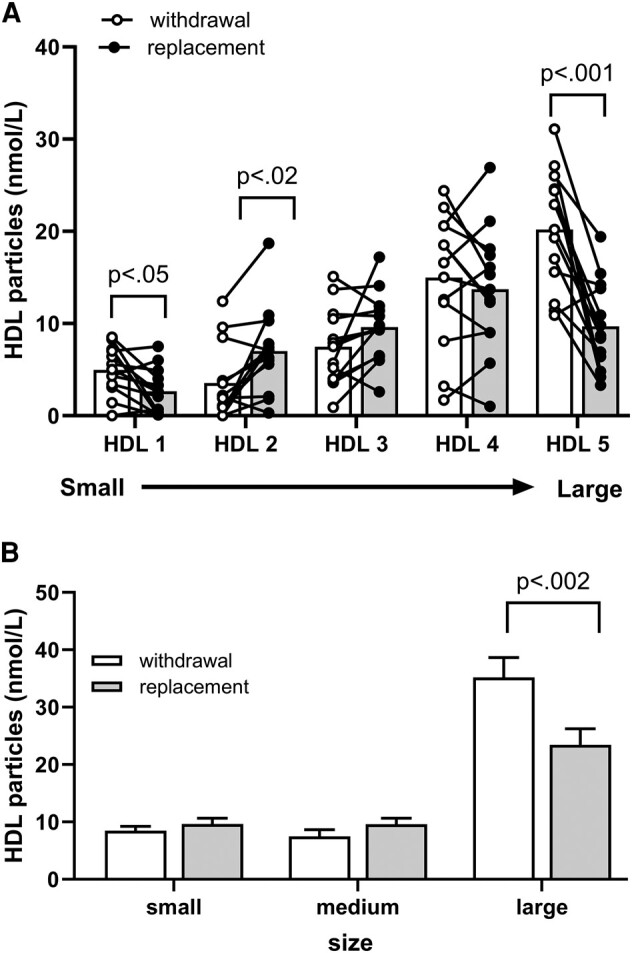
Effects of thyroid hormone withdrawal (open bars) and replacement (shaded bars) on high-density lipoprotein subclasses measured by nuclear magnetic resonance spectroscopy (mean ± SD). A, Particle concentrations of lipoproteins were calculated for 5 subclasses. B, Combined subclass particle concentration into small, medium, and large sizes. Data were analyzed by paired *t* test with Holm-Sidak multiple test correction.

When the participants were hypothyroid, particle number nearly doubled in HDL-1 (+52%, *P* = .04), declined by one-half in HDL-2 (−50%; *P* = .02) and mid-sized HDL-3, and increased 2-fold in the very largest HDL-5 (*P* = .0009) particles (Fig. 4A and Table 2). All the aforementioned changes were reversed when thyroid hormone was replaced.

### Comparing Individual and Combined Lipoprotein Subclasses Very Low-Density Lipoprotein and Low-Density Lipoprotein

The contours formed by the increments in particle number in the VLDL subclasses displayed individually and by size when thyroid hormone was withheld were identical (Fig. 1A and 1B). Since LDL has only 3 subclasses, it is already separated by size into small-, medium-, and large-sized particles (see Fig. 2).

### High-Density Lipoprotein

The J-shaped configuration formed by the changes in the individual HDL subclasses when the participants were hypothyroid (Fig. 4A) differs from the hockey stick configuration of the particle numbers in the subclasses grouped by size (Fig. 4B). Combining the HDL subclasses obscures the opposing directional changes of HDL-1 and HDL-2 that occurred when thyroid hormone was withheld (Fig. 4A and 4B). Similarly, merging the HDL- 4 and HDL-5 subclasses into a single category of large particles masks the fact that the observed elevation is attributable to an increase in HDL-5 and HDL-4 was essentially unchanged (see Fig. 4A and 4B).

### Particle Size

When the participants were hypothyroid, the average HDL particle size increased significantly (*P* = .0001; see Table 2). No change was observed in the size of VLDL or LDL.

## Discussion

In the present study in hypothyroid individuals, we demonstrate a close relationship between reductions in the activities of the thyroid hormone–dependent HDL remodeling proteins (LPL, CETP, HL, PLTP, LCAT) [14] and directional changes in particle number that are subclass specific. Importantly, the quantitative approach we have taken to expressing NMR-measured lipoprotein subclasses reveals that these distinctive physiologic associations have been obscured in previous studies in similar individuals because the 5 HDL subclasses have been combined according to their size and reported as 3 classes of small, medium, and large particles.

In the clinical setting of thyroid hormone deficiency, the principal reason for the elevation of plasma lipids and the smooth contours of the individually displayed increases of the apoB-containing VLDL and LDL subclasses is a delay in their removal from the circulation owing to diminished intravascular processing of TGRLPs into LDL by LPL, CETP, HL, and PLTP and in the activity of hepatic apoB, E LDL receptors that mediate their egress from plasma [14]. A number of other thyroid hormone–responsive processes are also likely to contribute to the altered plasma lipid profile in hypothyroid patients. These include increases in apoB synthesis [15] and levels of angiopoietins 3 and 8 [16] that inhibit LPL, and hepatic PCSK9 activity [17] that reduces the number of apoB, E receptors available for the clearance of apoB-containing lipoproteins from the circulation, and evidence that elevated TSH levels have a thyroid hormone–independent elevating effect of lipid panels [18].

The J-shaped profile formed by changes in particle number in the individual HDL subclasses contrasts sharply with the pattern formed by the increments in VLDL, LDL, and IDL when thyroid hormone was withdrawn. Their differing contours reflect the fact that the remodeling of apoB-containing lipoproteins and HDL in plasma are very dissimilar processes. The intravascular processing of apoB lipoproteins, for example, is unidirectional and involves the progressive, lipolytic downsizing of large TGRLP particles into much smaller LDL and their exit via apoB, E receptors [19].

In contrast, HDL transport in plasma is inherently more complex because it is bidirectional. On the one hand, it involves the generation and “upsizing” of new small HDL particles formed by apoA-1, LCAT, and products of CETP and PLTP action into HDL- 2 and mid-sized HDL-3 with CE [20] generated by LCAT activity and, on the other hand, the simultaneous reduction in size of mature HDL particles (HDL-4, HDL-5) resulting from the removal of core lipid TG acquired from VLDL during CET by HL and EL [21].

In light of the thyroid hormone dependence of the HDL remodeling proteins, it is not surprising that when its availability was severely limited that levels of the LCAT substrate HDL-1 increased and its products, HDL-2 and HDL-3, both declined. This precursor-product relationship is inapparent when HDL-1 and HDL-2 are combined into a single “small” HDL particle [3]. Similarly, merging HDL-4 and HDL-5 into “large” HDL particles masks the fact that this elevation is primarily attributable to an increase in HDL-5, a substrate for HL and EL [3].

While elevated LDL and IDL levels are believed to increase cardiovascular risk in overtly hypothyroid patients [22, 23], our measurements of the VLDL subclasses imply that disturbances in TGRLP transport also contribute to atherogenesis in the hypothyroid state. Specifically, as previously reported in the hypothyroid cohort by Pearce et al [3], we also found that our participants manifested an elevation of the VLDL-6, VLDL-5, and HDL-1 subclasses that has been shown to be associated with an increase in cardiovascular risk in euthyroid individuals with prediabetes [24], overt type 2 diabetes [25, 26], and in nondiabetic patients with coronary disease [27]. These observations in different populations are consistent with multiple studies demonstrating that TGRLPs are independent risk factors for atherosclerotic cardiovascular disease and evidence showing conclusively that TGRLPs are atherogenic [28].

Numerous population studies have shown that plasma concentrations of HDL-C and apoA-1, the major structural component of HDL, are independent inverse predictors of cardiovascular risk [29]. For this reason, it is noteworthy that overtly hypothyroid patients are at increased risk for coronary heart disease despite having elevated HDL-C and apoA-1 levels [13]. This finding suggests that when thyroid hormone is deficient, one or more of HDL's cardioprotective properties may be compromised. Among the many salutary functions of HDL such as facilitating the efflux of cholesterol from tissues and delivering it to SR-B1 receptors in the liver, inhibiting lipoprotein oxidation, thrombosis, and vascular inflammation, and promoting the integrity of endothelium [30], cholesterol efflux is the only metric that has been studied in hypothyroid individuals to date and found to be impaired [31].

We did not find that the number of atherogenic small dense LDL (sdLDL) in the LDL-1 subclass was increased as described previously in hypothyroid individuals [32]. The absence of sdLDL in our patient sample is likely because conditions favoring their formation were not present. Though their plasma TGs increased when thyroid hormone was withheld, the level reached was still in the normal range. For this reason, it is likely that that they lacked both sufficient numbers of apoC-III–containing TGRLP precursor particles [33] and the lipolytic actions of LPL and HL required for their conversion to sdLDL [34].

The strength of this study is that it demonstrates that when HDL subclasses are measured individually by NMR in hypothyroid individuals, directional changes in subclass particle numbers become apparent that correlate closely with the known sites of action of thyroid hormone–dependent lipoprotein remodeling proteins on each subclass. Functional relationships of this type probably have been obscured in past studies when measurements of the 5 HDL subclasses have been expressed as small, medium, and large particles.

These findings suggest that, when NMR is employed to examine the mechanisms of action of future lipid-modifying therapies, each HDL subclass should be evaluated individually because it provides a window into HDL transport and the activities of the HDL remodeling proteins that are masked when the HDL subclasses are combined according to size. One limitation of this study is that activities of the lipid-modifying proteins were not measured and the inferences we have drawn regarding changes in their activities in the hypothyroid state are based on previously published information [14]. Consequently, we are unable to correlate their activities with the magnitude of changes in particle numbers within each HDL subclass when the participants were hypothyroid. Another limitation is that we were unable to correlate individual TSH and total T4 measurements in our small study group with the quantitative changes in the HDL subclasses as Pearce et al [3] have done previously for the VLDL and LDL subclasses in a larger patient population.

## Data Availability

Some or all data sets generated and analyzed during the current study are not publicly available but are available from the corresponding author upon reasonable request.

## Abbreviations

apoB: apolipoprotein B
CET: cholesteryl ester transfer
CETP: cholesteryl ester transfer protein
EL: endothelial lipase
HDL: high-density lipoprotein
HL: hepatic lipase
IDL: intermediate-density lipoprotein
LCAT: lecithin cholesterol acyltransferase
LDL: low-density lipoprotein
LPL: lipoprotein lipase
NMR: nuclear magnetic resonance spectroscopy
PLTP: phospholipid transfer protein
sdLDL: small dense low-density lipoprotein
T4: thyroxine
TC: total cholesterol
TGs: triglycerides
TGRLPs: triglyceride-rich lipoproteins
TSH: thyrotropin
VLDL: very low-density lipoprotein
